# MicroRNA inhibition fine-tunes and provides robustness to the restriction point switch of the cell cycle

**DOI:** 10.1038/srep32823

**Published:** 2016-09-09

**Authors:** Ricardo C. H. del Rosario, Joseph Ray Clarence G. Damasco, Baltazar D. Aguda

**Affiliations:** 1 Stanley Center for Psychiatric Research, Broad Institute of MIT and Harvard, Cambridge MA 02142, USA; 2 Institute of Mathematics, University of the Philippines, Diliman, Quezon City 1101 Philippines; 3 Philippine Genome Center, University of the Philippines, Diliman, Quezon City 1101 Philippines

## Abstract

The restriction point marks a switch in G1 from growth factor-dependent to growth factor-independent progression of the cell cycle. The proper regulation of this switch is important for normal cell processes; aberrations could result in a number of diseases such as cancer, neurodegenerative disorders, stroke and myocardial infarction. To further understand the regulation of the restriction point, we extended a mathematical model of the Rb-E2F pathway to include members of the microRNA cluster miR-17-92. Our mathematical analysis shows that microRNAs play an essential role in fine-tuning and providing robustness to the switch. We also demonstrate how microRNA regulation can steer cells in or out of cancer states.

The Restriction Point (R) is a checkpoint in G1 that marks the transition from growth factor-dependent to growth factor-independent cell cycle progression^1^. Most human cancers exhibit dysfunctional R^2^,^3^,^4^,^5^, with many of the known genes involved in its regulation acting as either oncogenes or tumor suppressor genes (Fig. 1).

R is marked by an abrupt accumulation of the pro-proliferative members of the family of transcription factors E2F (namely, E2F1, E2F2 and E2F3) that induce expression of genes required for DNA replication^6^,^7^,^8^. Using fluorescent reporters for E2F transcriptional activity in single cells, Yao *et al.*^9^ provided the first *in-vitro* demonstration that R acts as a bistable switch–as predicted by the authors’ own analysis of a minimal model of the Rb-E2F pathway that involves positive feedback loops.

In a related study, Aguda *et al.*^10^ considered an autocatalytic protein module (composed of E2F and c-Myc) coupled with the microRNA cluster miR-17-92 via a negative feedback loop (*e.g.*, E2F1 inducing expression of miR-17-92, and members of the latter inhibiting E2F1). The 2-variable model analyzed by Aguda *et al.*^10^ – henceforth referred to as the ‘A model’ – elegantly predicted the role of miR-17-92 in tuning the sequence of cellular states, from quiescence to proliferation and to apoptosis. Furthermore, it was postulated that between normal cell proliferation and onset of apoptosis is a range of oncogenic activity (of E2F1 or c-Myc) with significant probability of the cancer state; such a range is referred to as the ‘cancer zone’^10^.

We extended the model of the Rb-E2F pathway analyzed by Yao *et al.*^9^ to include miR-17-92. This Rb-E2F pathway could generate a resettable bistable switch^11^, with the kinetic parameters used by Yao *et al.*^9^ fitted with their experimental data. The model analyzed in this paper – which we call the R model (shown in Fig. 2) – provides a framework for analyzing the influence of miR-17-92 on the properties of the restriction point switch. We show that, as the microRNA inhibition efficiency is varied, the influence of miR-17-92 on the characteristics of the bistable switch of the R model match those of the A model. Furthermore, by showing that the R model allows for a comparative analysis of microRNA regulation of c-Myc and E2F, we show that the R model offers predictions beyond those made by the A model.

There is growing evidence that microRNAs play an important role in the development and progression of various diseases^12^,^13^,^14^,^15^,^16^,^17^. In this paper, we are interested in the role of microRNAs in conferring robustness to biological processes^18^,^19^,^20^,^21^,^22^. One mechanism by which microRNAs perform this function is by buffering against noise in gene expression. Previous studies have investigated the buffering effect of noise by various microRNA canonical network motifs: positive and negative feedback^23^,^24^,^25^ and feed-forward loops^26^,^27^. The network of the R model (Fig. 2b) includes all these canonical network motifs. First, c-Myc and the microRNA cluster form an incoherent feed forward loop in regulating E2F. Second, E2F induces a positive feedback on itself. Third, the microRNA cluster induces a negative feedback on c-Myc and on E2F. And finally, c-Myc and E2F form a coherent feed forward loop in regulating the microRNA cluster. Thus, the R model provides insights into microRNA buffering of noise that is more comprehensive than analyzing individual canonical network motifs. The R model could therefore help in further understanding the design principles underlying biological robustness.

## Results

### The R model is bistable

**Figure 2:**
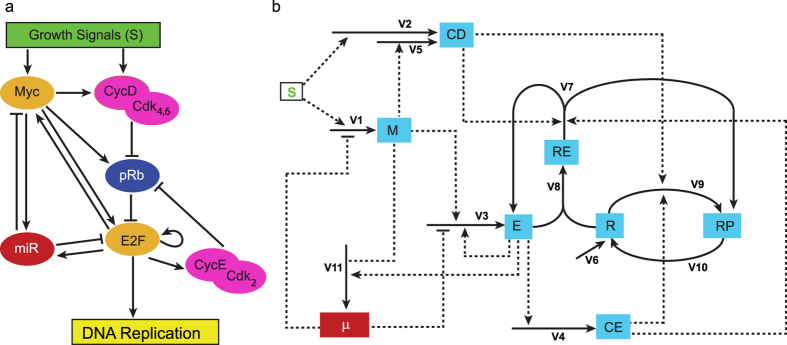
The network of the R model. (**a**) Schematic diagram of Rb-E2F network. Arrows depict activation or upregulation; hammerheads mean inhibition or downregulation. miR refers to some members of the miR-17-92 microRNA cluster. Gene names are the same as in Fig. 1.(**b**) Detailed network of the R model showing the model variables (boxes) and reaction steps (solid arrows). The labels of the solid arrows correspond to the reaction rates in Eqs (1 and 2) (Methods). Dashed lines indicate which variables affect the reaction rate. The degradation steps for all the nodes in the network are not shown in the diagram but are considered in the kinetic equations (Methods). Abbreviations: *M* = c-Myc, *E* = E2F family of transcription factors (E2F1, E2F2 and E2F3), *CD* = cyclin D, *CE* = cyclin E, *R* = retinoblastoma protein (pRb), *RP* = phosphorylated pRb, *RE* = pRb-E2F complex, *μ* = miR-17-92 cluster, *S* = growth signals.

The R model is a mathematical representation of the network of interactions of Fig. 2 (see Methods). It is an extension of the model in Yao *et al.*^9^ with additional reactions involving the interactions between the miR-17-92 cluster and the Rb-E2F pathway. These new interactions include the inhibition of E2F by miR-17-92, which is well known^8^,^28^, and the inhibition of Myc by the miR-17-92 cluster, which was recently shown to be via an indirect manner^29^. Chek2 was found to be a target of miR-17-19b, and down-regulation of Chek2 leads to increased recruitment of HuR/RISC to MYC mRNA, which inhibited Myc translation^29^.

**Figure 3:**
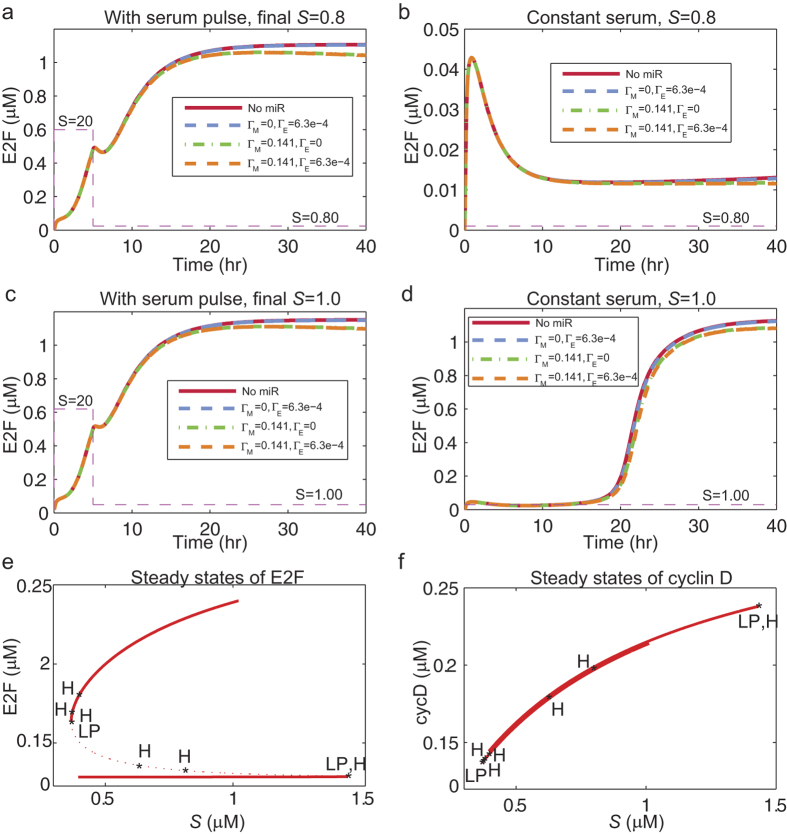
History dependence of the R model and its bistable states. **(a)** E2F concentrations of a system with a serum pulse (*S* = 20 *μ*Μ) that lasts 5 hours and then drops to a final level of *S* = 0.8 *μ*Μ. **(b)** System without a pulse and with a constant serum level of *S* = 0.8 *μ*Μ. **(c)** and **(d)** are similar to (**a**,**b**), respectively, except that the final/baseline serum level is at a higher level of *S* = 1.0 *μ*Μ. The different lines in (**a**–**d**) represent different microRNA inhibition strengths (legend). **(e**,**f)** Equilibrium plots of E2F and cyclin-D showing saddle-node (LP) and Hopf (H) bifurcation points. Solid lines: stable steady states; dotted lines: unstable steady states.

We hypothesized that the R model, like the Yao model, is bistable. To verify this hypothesis, we simulated a pulse experiment where a serum pulse was used to stimulate serum-starved and quiescent cells. Yao *et al.*^9^ used this experiment to show the history dependence of E2F expression on serum concentration. When a serum pulse of *S* = 20 *μ*M was introduced for 5 hours and then was dropped to *S* = 0.8 *μ*M, the E2F values proceeded to the on state even after the serum pulse was removed (Fig. 3a; the on state is E2F > 1.0 *μ*M). In the absence of a serum pulse, E2F levels stayed within the quiescent zone or off state (Fig. 3b; the off state is E2F < 0.01 *μ*M). These results demonstrate the history dependence of the R model. We repeated the simulations using a higher final serum level of *S* = 1.0 *μ*M, and for the case with a serum pulse, the model behaved similarly as before (Fig. 3a,c). However, when there was no serum pulse, the system switched to the on state (Fig. 3b,d). This history dependence of the R model is a hallmark of a bistable switch.

The bistability of the R model can be analyzed by determining the stability of its equilibrium points (Methods). By calculating the equilibrium curve of the model variables with *S* as a parameter and all other parameters fixed (Fig. 3e,f), we found that the equilibrium curve of E2F had similar properties to those of the equilibrium curve of protein *p* of the two-dimensional A model^30^. The curve was “S-shaped” and had a region in which the system had two stable steady states (Fig. 3e). The equilibrium curve of cyclin-D also included a region where the system had two stable steady states, but the two states were too close to each other making them distinguishable (Fig. 3f). This agrees with experimental observations where the histogram of single cell measurements of cyclin-D did not show a bimodal distribution^9^.

We also hypothesized that the R model has an excitable state. This is a state where, the input pulse can drive the system to the on state, but due to the insufficiency of either the magnitude or time duration of the serum pulse, the system returns to the off state after a short period. The A model has been shown to have an excitable state^30^ and the R model also exhibited this property (Supplementary Fig. 1).

For a system with relatively stronger microRNA inhibition (Fig. 3a–d, *orange* and *green* lines), the E2F values at steady state deviated from those of the plots with no or weak microRNA inhibition (Fig. 3a–d, *red* and *blue* lines). This shows one manner in which the microRNA cluster can fine-tune the Rb-E2F network, which will be analyzed in detail below.

### MiR-17-92 feedback inhibition confers robustness to the Rb-E2F pathway

**Figure 4:**
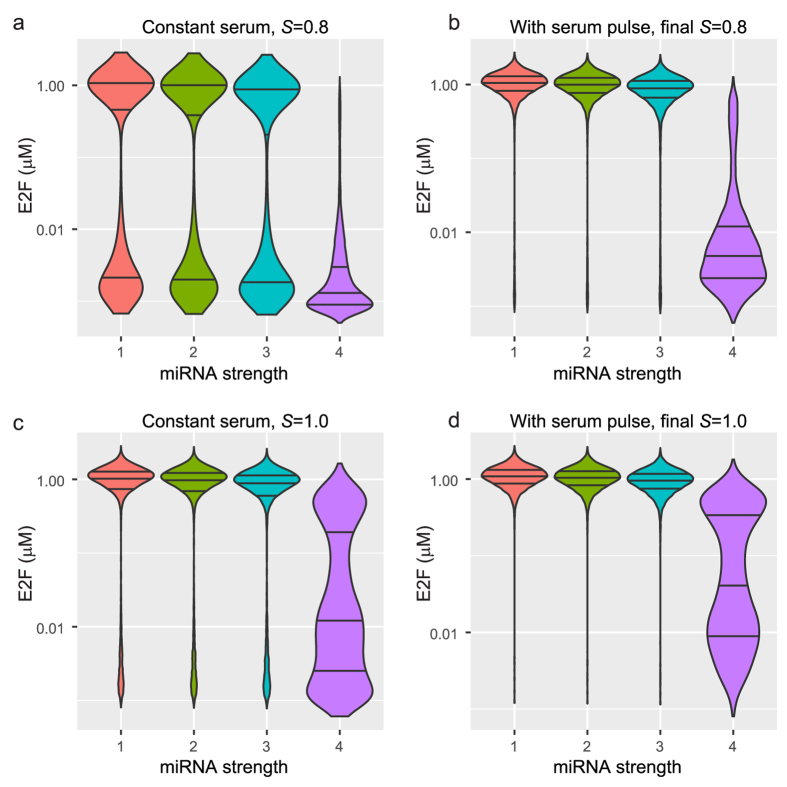
Noise buffering by microRNAs. For specific values of the microRNA inhibition parameters *Γ*_E_ and *Γ*_M_, stochastic simulations of 10,000 cells were performed and the violin plots of the values of E2F at *t* = 100 hr were plotted. **(a)** Constant serum level of *S* = 0.8 *μ*M. **(b)** Serum pulse of *S* = 20 *μ*M that lasts for 5 hours and final serum level of *S* = 0.8 *μ*M. **(c**,**d)** are similar to (**a**,**b**), respectively, except that the constant/final serum level was 1.0 *μ*M. The *x*-axis indicates microRNA inhibition strength ordered in increasing values: 1, orange: *Γ*_E_ = *Γ*_M_ = 0 (no microRNA); 2, green: *Γ*_E_ = 6.3e-4, *Γ*_M_ = 0.0404; 3, cyan: *Γ*_E_ = 6.3e-4, *Γ*_M_ = 0.141; 4, purple: *Γ*_E_ = 6.3e-4, *Γ*_M_ = 1.8. For each violin plot, horizontal lines indicate the 25, 50 and 75 percentiles (Supplementary Table 2). The values of other parameters are given in Table 1. The number of cells in the off and on states are given in Supplementary Table 1.

A population of cells synchronized at the same cell cycle phase can exhibit cell-to-cell variation due to stochastic events. Within the Rb-E2F pathway, these stochastic events could come from mRNA degradation or translation, or from cell-to-cell differences in protein concentrations, or other environmental factors. We incorporated these stochastic effects by adding noise terms to the R model and simulated the model within a population of 10,000 cells (Methods). For a cell population with a constant serum of *S* = 0.8 *μ*M, the E2F levels exhibited a bimodal distribution when microRNA inhibition was absent or had low strength (Fig. 4a, *orange*, *green*, and *cyan* plots; Supplementary Tables 1 and 2). One mode was at the off state (around E2F < 0.01 *μ*M) and another at the on state (around E2F = 1.0 *μ*M). In the non-stochastic simulations, the constant serum level of *S* = 0.8 *μ*M was not enough to switch the cells to the on state (Fig. 3b), hence the bimodal distributions in Fig. 4a indicate that noise within the pathway could cause the R switch to turn on in some cells. When microRNA inhibition was strong enough, the distribution was unimodal at the off state (Fig. 4a, *purple* plot), indicating that this microRNA inhibition strength was effective in guarding against stochastic switching.

When the constant serum level was high (*S* = 1.0 *μ*M), the stochastic simulations showed a bimodal distribution but with very low numbers at the off state (Fig. 4c, *orange, green*, and *cyan* plots). The strongest microRNA inhibition (Fig. 4c, *purple* plot) was not enough to keep the cells within the off state. This agrees with the result in Fig. 3d, where the cells switched to the on state when a constant serum level of *S* = 1.0 *μ*M was applied even if there was no serum pulse.

Next we repeated the stochastic simulation experiment but this time provided a serum pulse (*S* = 20 *μ*M for the first 5 hours, Fig. 4b,d). At a final serum level of *S* = 0.8 *μ*M, we expected the pulse to drive all cells to the on state (see Fig. 3a), but noise can prevent some of the cells from switching on (Fig. 4b, *orange, green,* and *cyan* plots show a few cells at the off state; Supplementary Tables 1 and 2). However, the strong microRNA inhibition counteracted the memory dependence of the system (on the serum pulse) since most of the cells did not switch on (Fig. 4b *purple* plot). When the final serum level was *S* = 1.0 *μ*M, almost all cells switched on even with weak microRNA inhibition (Fig. 4d, *orange, green,* and *cyan* plots), but strong microRNA inhibition was able to keep a significant amount of cells in the off state (Fig. 4d, *purple* plot; Supplementary Tables 1 and 2).

To further understand the effect of microRNA regulation on the robustness of the system, we performed an analysis on how it affects noise amplification and noise susceptibility (Methods). Noise amplification measures the ratio between the noise in the output (the model output we consider is E2F and noise is defined as the coefficient of variation) and the noise in the input (coefficient of variation of *S*). On the other hand, noise susceptibility measures the relative change in the model output (E2F) in response to minute changes in the input (*S*). A previous analysis of noise amplification and susceptibility on the A model has shown that these two robustness measures had properties that depended on whether the system was at the on state or at the off state^31^.

**Figure 5:**
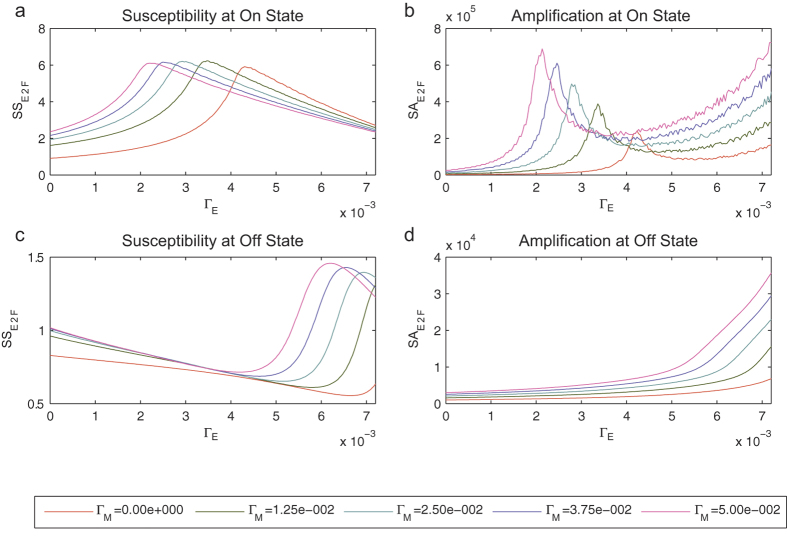
Noise amplification (*SA*) and noise susceptibility (*SS*) curves for variable E2F as parameters *Γ*_E_ and *Γ*_M_ vary. 5 different values of parameter *Γ*_M_ (legend bar) were used and the values of the other parameters are given in Table 1. (**a**) Noise susceptibility at the on state. **(b)** Noise amplification at the on state. **(c)** Noise susceptibility at the off state. **(d)** Noise amplification at the off state. Initial conditions that lead to the on and off states were chosen, and the model values at the steady states were used in calculating noise amplification and susceptibility (Methods). Results using different pairs of parameters are given in Supplementary Figs 2–6.

Here, we analyze three parameters, the microRNA inhibition parameters *Γ*_E_ and *Γ*_M,_ and the positive feedback parameter *k*_*E*_, instead of two parameters analyzed in ref. 31 for the A model. We plotted results for two parameters at a time, keeping the values of the other parameters fixed (Fig. 5 and Supplementary Figs 2–6). In Fig. 5, the noise amplification values (*SA*) and noise susceptibility values (*SS*) were plotted for each value of *Γ*_E_ and *Γ*_M_, with *Γ*_E_ on the *x*-axis and with each plotted line corresponding to a value of *Γ*_M_. The noise amplification of variable E2F at the on state showed a local maximum with respect to inhibition parameter *Γ*_E_, and trended upward after the peak (Fig. 5b). At the off state, the noise amplification monotonically increased as *Γ*_E_ increased (Fig. 5d). Thus there is a qualitatively different effect of microRNAs on noise amplification between the on and off states. We obtained the same qualitative difference between noise amplification at the on and off states when we plotted *Γ*_M_ on the *x*-axis (Supplementary Fig. 2).

When we paired the positive feedback parameter *k*_*E*_ with either *Γ*_E_ or *Γ*_M_, we obtained an opposite effect at the off state where the noise amplification curves decreased with increasing values of *k*_*E*_ (Supplementary Figs 3–6). This demonstrates the opposing effects of the positive activation and negative inhibition parameters on noise amplification.

The noise susceptibility curves showed peaks at both on and off states (Fig. 5a,c) and one obvious qualitative difference between the two is that at the on state, the value of *Γ*_E_ at which the peak occurs decreases as *Γ*_M_ increases, while the opposite holds at the off state. This also seems to be true when *Γ*_M_ was plotted on the *x*-axis (Supplementary Fig. 2).

### MiR-17-92 fine-tunes the Rb-E2F pathway in and out of the cancer zone

**Figure 6:**
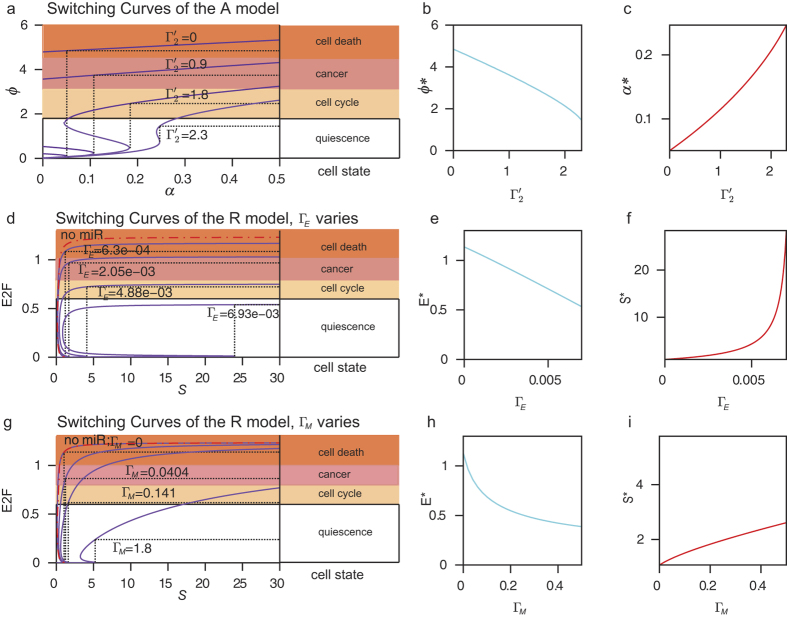
Fine-tuning of the R switch by microRNAs. **(a)** Switching behavior of the A model with respect to parameters *Γ*_2_′ and *α*′. For a given value of *Γ*_2_′, the equilibrium curve of *ϕ* is plotted with respect to *α*′ (*purple* lines). At the saddle point (vertical lines), the higher stable steady state value of *ϕ* is the “on” value denoted by ϕ* and the value of *α*′ is the “off-on” value denoted by α*. The value of ϕ* determines the ensuing state of the cell, whether it will go into quiescence, cell cycle, cancer, or apoptosis. Cell states are indicated by the color of the regions. **(b)** and **(c)** The parameter *Γ*_2_′ of the A model controls both α* and ϕ*. Fixed parameters: *ε* = 0.1, *κ* = 5, *Γ*_1_′ = 1. **(d)** and **(g)** are similar to (a) except that the analysis was done on the R model and two parameters were considered: *Γ*_E_ (**d**) and *Γ*_M_ (**g**). The saddle node denotes the switching point (S*, E*), which is analogous to (α*, ϕ*) of (**a**). **(e**,**f)** The parameter *Γ*_E_ can simultaneously control S* and E*. **(h**,**i)** The parameter *Γ*_M_ controls both S* and E*. The values of the rest of the R model parameters are given in Table 1.

There are two experimentally observable variables associated with the ‘all or nothing’ switching behavior of the bistable restriction point switch. One variable is the value of the serum growth factor where the switch occurs, and the other is the magnitude of the jump by the E2F concentration to the on value (Fig. 6a). The significance of the magnitude of the on jump, as discussed in Aguda *et al.*^8^, is that it determines whether the cell remains quiescent, proliferates, enters the cancer zone, or undergoes apoptosis. In the previous sections, we considered a coarse grained classification of the values of E2F, with E2F < 0.01 being off, and E2F > 1.0 being on. Now, we subdivide the on state into the following cell states: cell cycle, cancer, or apoptosis.

We first extended the switching behavior analysis of the 2-dimensional A model to explore other possible model predictions that may carry over to the more complex R model. The parameters of interest in the A model are the microRNA inhibition parameter (*Γ*_2_′) and the rate of protein production stimulated by the growth serum (*α*). The off-on value is symbolized by α*, while the magnitude of the on jump is symbolized by ϕ* (Fig. 6a). When there was no microRNA inhibition (*Γ*_2_′ = 0), the on jump was extremely large, resulting in cell death, even at very small values of α* (Fig. 6a). This indicates that the initiation of proliferation could be at the mercy of noise (again, this means no control of proliferation). Figure 6b,c show that as the microRNA inhibition parameter *Γ*_2_′ increases, ϕ* decreases while α* increases, indicating that varying *Γ*_2_′ hits both essential switching features (α* and ϕ*) simultaneously to the “right” values, (i.e., *Γ*_2_′ can be used to decrease ϕ* to fall below the cancer region). Thus, α* and ϕ* are coupled, with an inverse relationship, and are optimally controlled by microRNA via the parameter *Γ*_2_′.

We performed an analogous analysis for the R model using the microRNA parameters *Γ*_M_ and *Γ*_E_. The experimentally observable variables associated with the all-or-nothing switching behavior of the R model are S* and E*, which correspond respectively to the switch variables α* and ϕ* of the A model. The protein we observe for switching, as in the A model, is E2F since its concentration determines whether the switch will turn on or off. We found out that similar to the A model, when there was no microRNA inhibition (Fig. 6d,g dashed lines), the on-off value for the serum level, S*, was relatively small, while the on value E* jumps to the highest level, indicating that the cell fate of either normal proliferation or cell death is susceptible to noise. We also found that, similar to the A model, as the microRNA parameters (*Γ*_M_ or *Γ*_E_) increase, S* increases while E* decreases (Fig. 6e,f,h,i). However, the rates of increase of S* and decrease of E* are qualitatively different from the A model. This indicates that by varying its rate of inhibition on either *E* or *M*, or both, the microRNA machinery has enough flexibility to fine-tune the R switch and to provide robustness against noise.

A comparison of Fig. 6e,h shows that *Γ*_M_ lowers the on value E* faster than *Γ*_E_, suggesting that this parameter could be more useful in controlling the robustness of the system against noise. The inverse relationship between the microRNA inhibition efficiency and the on value agree with the numerical simulations of an engineered microRNA circuit which showed that a stronger microRNA repression corresponds to a smaller difference between the off and on states of the protein expression levels^23^. Thus, Fig. 6 highlights the importance of the microRNA machinery in steering the cell fate by lowering the on value.

### The R model is robust to changes in the microRNA associated parameters *Γ*_E_ and *Γ*_M_

Now that we have gained some insights on how the miR-17-92 cluster can fine-tune the expression of its target genes in the Rb-E2F pathway, we ask how sensitive the Rb-E2F pathway is with respect to changes in the microRNA parameters *Γ*_M_ and *Γ*_E_. Note that in the noise susceptibility analysis, we quantified the sensitivity of each variable at steady state to the input *S*. Here, we quantified the sensitivity of the whole system to a particular parameter over a time interval (Methods).

The time-dependent sensitivity of the R model to a parameter *p*_i_ depends on nominal parameter values (Methods). We chose 3,168 parameter sets from the various values of the parameters we used in numerical simulations (Supplementary Table 4; note that only the parameters *S*, *Γ*_M_, *Γ*_E_ and *k*_E_ were varied in our analysis) and calculated the time-dependent sensitivity (Eq. 10) for each set. We plotted the sensitivity values for each of the 33 parameters in Supplementary Fig. 7. The system did not show a high sensitivity to parameters *Γ*_M_ and *Γ*_E_ relative to the other parameters, indicating that microRNA regulation does not introduce drastic changes to the R switch. Furthermore, the relatively low sensitivity of the system to perturbations in these two parameters indicates that the system is robust with respect to small changes in these parameters.

### Bifurcation analysis shows that the R model has complex dynamical properties

**Figure 7:**
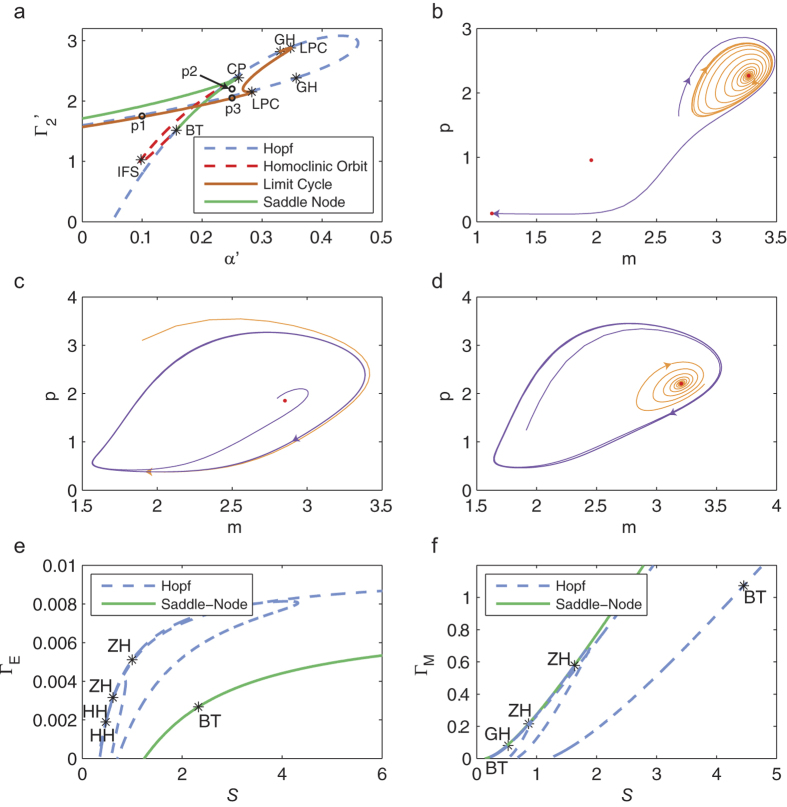
Bifurcation analysis of the A and R models. **(a)** In addition to the previously discovered saddle-node (*green line*) and Hopf bifurcation curves (*blue dashed lines*) of the A model^30^, we discovered a limit cycle curve *(brown line*) and a homoclinic orbit (*red dashed line*). We also discovered a Bogdanov-Takens bifurcation point (BT) which is a bifurcation of co-dimension 2. **(b–d)** Phase portraits using parameter values indicated by *p*_1_, *p*_2_, and *p*_3_, respectively, in (**a**). Red circles indicate equilibrium points and for each panel, two representative phase portraits illustrate the stability of the equilibrium points and limit cycles. **(e)** Two-parameter bifurcation analysis of the R model on the parameters *S* and *Γ*_E_. **(f)** Two-parameter bifurcation analysis of the R model on the parameters *S* and *Γ*_M_. Abbreviations: GH = Generalized Hopf, LPC = Limit Point Cycle, CP = Cusp Point, BT = Bogdanov-Takens, IFS = Inclination-flip with respect to the stable manifold, LP = saddle-node, H = Hopf, HH = double Hopf, ZH = zero-Hopf. For the A model, the fixed parameter values were: *ε* = 0.1, *κ* = 5, *Γ*_1_′ = 1, while for the R model, the fixed model parameters are given in Table 1.

We performed bifurcation analysis on the R model (Methods) and discovered a co-dimension 2 bifurcation – a Bogdanov-Takens bifurcation, together with global bifurcations involving limit cycles (Fig. 7a). The Hopf and saddle-node curves of the A model have been previously reported in Li *et al.*^30^, where they delineated the bulk diagram of the dynamical behavior of the A model into four regions, namely monostability, bistability, excitability, and undamped relaxation oscillation. Our discovery of global bifurcations provides a finer decomposition of this bulk diagram. Point *p*_1_, which is between the brown and dashed red curves of Fig. 7a, is within the bistable region of Li *et al.*, but one of the stable equilibrium points lies inside an unstable limit cycle (Fig. 7b). At point *p*_2_, the system has an unstable equilibrium point inside a stable limit cycle (Fig. 7c), and at point *p*_3_, the system has a stable equilibrium point inside a stable limit cycle (Fig. 7d). The complexity of the dynamics of the A model underscores the importance of using a coarse-grained model as it can still exhibit surprising complex dynamical properties.

To plot the analogous bifurcation curves for the R model, we performed two sets of analysis, one with parameters *S* and *Γ*_E_ (Fig. 7e) and another with parameters *S* and *Γ*_M_ (Fig. 7f). As in the A model, we also found Hopf and saddle-node curves, but these curves delineated the parameter space into more regions than in the A model. We also found Bogdanov-Takens bifurcations (Fig. 7e,f).

## Discussion

Stochastic simulations illustrating noise buffering of microRNAs at low serum levels (Fig. 4) support currently held ideas that microRNAs appear to be non-essential under normal conditions, but are essential under environmental and genetic perturbations^20^,^32^. Furthermore, these results are consistent with experimental observations based on an engineered microRNA circuit which showed that the presence of a microRNA-mediated feedback loop consistently conferred robustness to the positive feedback loop motif, allowing the cells to maintain their off state for a longer time^23^. Moreover, the importance of the microRNA cluster in regulating the Rb-E2F pathway is also emphasized by previous results that the use of transcriptional repressor instead of microRNAs was not as effective in dampening fluctuations in the output of the target protein^27^,^33^. Aside from robustness to noise, the R model has also been shown to be robust with respect to perturbations on the connectivity of the network^34^. Our time-dependent sensitivity analysis showed that the whole system is robust with respect to small changes of the microRNA inhibition parameters. Taken together, our results and those of others indicate that microRNAs play an essential role in providing phenotypic robustness to the Rb-E2F pathway.

The noise-buffering role of the miR-17-92 cluster aids the R switch in correctly processing the input signal, and demonstrates its important role in the decision process within the cell cycle^28^,^35^. Our analysis of the switching characteristics of the model with respect to the microRNA inhibition parameters emphasize the role of microRNAs in steering the cell away from the cancer zone by lowering the on value (Fig. 6). This result further provides a concrete framework for experimentally controlling the R switch: the experimentally observable quantities S* and E* can be controlled by varying the strength of the microRNA inhibition, which can be performed using synthetic biology.

The noise amplification curves showed a different qualitative behavior of noise amplification between the on and off states. Furthermore, the two microRNA parameters *Γ*_M_ and *Γ*_E_ displayed similar qualitative effects on noise amplification (Fig. 5b, Supplementary Figs 2, 5 and 6). This consistency is not surprising since although *Γ*_M_ does not directly inhibit E2F, its inhibition of c-Myc will decrease the positive influence of c-Myc on E2F. The effect of parameter *k*_*E*_ on noise amplification is opposite that of *Γ*_M_ and *Γ*_E_: at the on state, the peaks of the noise amplification curves move to the right (Supplementary Figs 3 and 4), instead of moving to the left (Supplementary Fig. 2) as *k*_*E*_ increases. Parameter *k*_*E*_ also had the opposite noise amplification effect at the off state: the noise amplification increases with increasing value of *k*_*E*_ (Supplementary Figs 3 and 4). These results are consistent with previous results in the analysis of the A model where it was shown that the positive feedback parameter κ and the negative feedback parameter *Γ*_2_ produced opposing effects on noise^27^. Thus, our results demonstrate that the insights obtained from network motifs can be extended to more realistic models. In particular, we have exhibited in the larger Rb-E2F network the result from an analysis of the A model^27^ that interlinked positive and negative feedback loops dynamically tune propagation signals.

Comparing properties of the R model with those of the A model, we were able to identify robust properties of the simple 2-D model that carry over to the more complex model. These properties are the switching curve characteristics, bifurcation properties, noise susceptibility, and noise amplification. The R model also presents insights that could not be gained from the A model. In particular, as mentioned above, the R model provides two parameters *Γ*_M_ and *Γ*_E_ that could be experimentally varied to determine how microRNAs control experimentally observable quantities S* and E*.

We anticipate that a similar role of microRNAs in conferring robustness could also be identified in other systems using the same analysis that we have performed. But we also anticipate that an analysis of a much larger miR-17-92 network, of which the Rb-E2F pathway is just a sub-network, will also be of interest in modeling the cell cycle. For example, a recent study showed that out of the thousands of genes controlled by Myc, the miR-17-92 cluster plays a special role in maintaining the neoplastic state of Myc-induced tumors^36^. The authors showed that Myc through miR-17-92 directly suppresses the expression of chromatin regulatory genes, which causes autonomous proliferation and self-renewal. For such larger networks, the analysis we have performed can be used for investigating the robustness of the system.

## Methods

### The R model

The R model is the mathematical representation of the dynamics of the network interactions in Fig. 2, and is given by the following system of differential equations


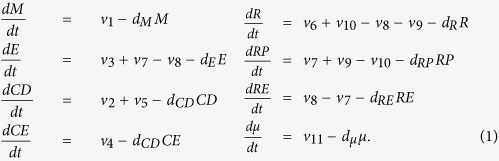


The model variables *M*, *E*, *CD*, *CE*, *R*, *RP*, *RE* and *μ* denote the concentrations, respectively, of c-Myc, E2F, cyclin-D, cyclin-E, retinoblastoma protein, phosphorylated retinoblastoma protein, retinoblastoma protein-E2F complex, and the miR-17-92 cluster. The terms *v*_i_ denote the reaction rate of the corresponding numbered reactions in Fig. 2b and are given by


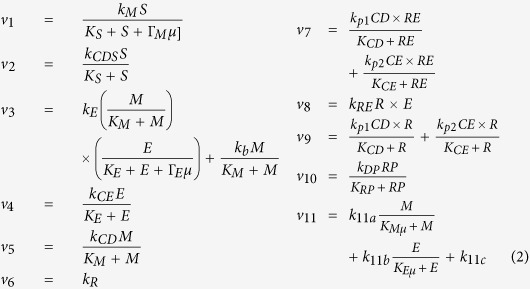


*S* is the concentration of the growth signal which will be used as the input in the analyses below, *Γ*_M_ is the inhibition coefficient of miR-17-92 on c-Myc, and *Γ*_E_ is the inhibition coefficient of miR-17-92 on E2F. The values of the parameters are given in Table 1.

The expressions above (Eq. (2)) are the same as in the Yao model^9^, except for the steps that embody microRNA inhibition, *v*_1_ and *v*_3_. The reaction rate *v*_1_ is the rate of c-Myc synthesis which phenomenologically models microRNA inhibition. The rate of E2F expression (*v*_3_) is given by two terms: the first term corresponds to the synergy between the transcription factors E2F and c-Myc, while the second term is due to c-Myc-induced transcription of E2F. The variable *μ* does not appear in the denominators of the expression involving *M* in *v*_3_ since those expressions model the positive regulation of E2F by c-Myc and thus are not directly affected by microRNAs (Fig. 2). Each model variable has a decay rate (with parameter *d* subscripted by the corresponding variable), which is given in the last term in each of the equations in Eq. (1). Note that although there are at least two mature microRNAs from the mir-17-92 cluster that target c-Myc and E2F, we assume here that these microRNAs are all represented by the single variable *μ*^8^.

To simplify notation, we write Eq. (1) concisely as


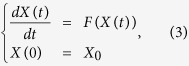


where *X(t)* is the vector with 8 components (*M*, *E*, *CD*, *CE*, *R*, *RP*, *RE*, *μ*), and *F(X*) is the right hand side of of Eq. (1). The initial conditions are given in Table 1.

Yan *et al.*^37^ extended the model of Yao *et al.*^9^ to include miR-449 inhibition. However, there are two fundamental differences between their model and our R model: miR-449 only inhibits c-Myc (and not E2F) and they modeled microRNA inhibition of c-Myc in a different manner. Thus, due to the differences in the network connectivity and model kinetics, the two models are qualitatively and quantitatively distinct.

The initial conditions and parameter values in Table 1 were taken from Yao *et al.*^9^, except for the microRNA parameters *d*_*μ*_, *K*_*Mμ*_ and *K*_*Eμ*_. We chose the value *d*_*μ*_ = 0.001 since microRNAs are assumed to be more stable than the mRNAs they regulate. The values of *K*_*Mμ*_ and *K*_*Eμ*_ were chosen to be slightly lower than those of *K*_*M*_ and *K*_*E*_. The various values of the parameters *S*, *Γ*_*E*_ and *Γ*_*M*_ used in the analyses are given either in the text or in figure captions.

The R model is provided as a Matlab function in Supplementary File 1. We also provide a Matlab script that uses the function to plot Fig. 3a–d.

### Stochastic simulations

To model stochastic effects, we added noise to the R model of the form


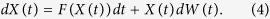


Here *W(t)* is the Brownian motion and *F(X(t))* is the right hand side of the system of ordinary differential equations from Eq. (1). To perform numerical simulations, we modified a C implementation of a 4^th^ order Runge-Kutta method for stochastic differential equations (http://people.sc.fsu.edu/~jburkardt/cpp_src/stochastic_rk/stochastic_rk.html)^38^. We used a noise level of 0.01 and a time step of 5e-4. For each set of parameters in Fig. 4, we performed 10,000 numerical integrations to simulate a clonal population of 10,000 cells. We recorded the values of the variables at *t* = 100. The initial conditions and parameter values are given in Table 1.

### Noise amplification and noise susceptibility

To analyze the effect of the microRNA inhibition parameters on the noise amplification and noise susceptibility of the R model, we used the Fluctuation Dissipation Theorem (FDT)^39^, with the serum *S* as the input to the system. The steady state susceptibility or noise sensitivity (*SS*) is defined as the relative change in the output in response to the change in the input


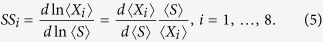


The subscripts denote the model variables, while the symbol 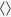 indicates the average value at steady state. We denote *S* by *X*_0_, and re-write the system of ODEs as


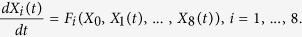


At steady state, the derivative of *F* with respect to *X*_*0*_, …, *X*_*8*_ is given by


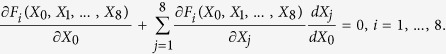


The system of equations above can be written as


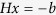


where


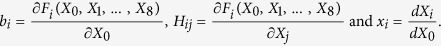


After solving for *x* from the linear system, the sensitivity to noise input, *SS*_i_ (Eq. 5) is obtained from


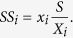


Note that for convenience, we dropped the use of the notation 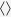.

On the other hand, the noise amplification (*SA*) is defined as the ratio between the output and input noise, with the noise defined as the coefficient of variation


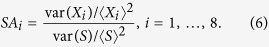


To compute the noise amplification *SA*_i_ (Eq. (6)), we form the 9 × 9 linear system that arises from the normalized FDT equation at steady state^39^,^40^


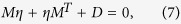


where


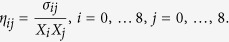


The components of matrices *M* and *D* are given in the Supplementary Material. For a given set of parameter values and initial conditions, we numerically solved the system of ODEs until steady state and then formed the FDT linear system (Eq. (7)). Then we numerically solved the linear system Eq. (7) for *η* using Matlab’s solver for Lyapunov equations lyap. The diagonal elements of *η* are the noise approximations (*σ*_i_^2^/*μ*_i_^2^) and Eq. (6) is calculated as


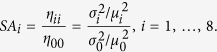


The range of the values of the parameters used in plotting Fig. 5 and Supplementary Figs 2–6 were dependent on the maximum value where the parameter space could still yield a bistable system (Supplementary Table 3).

### Switching characteristic analysis

In order to analyze how microRNAs fine-tune the Rb-E2F pathway in and out of the cancer zone^10^, we computed the values of the serum (*S*) at which the system jumps from one stable steady state to the other. To this end, we created a custom Matlab script that integrated the system until steady state and calculated the eigenvalues of the Jacobian at the steady state. The eigenvalues indicated the stability of the equilibrium point. An initial equilibrium point was obtained by running the Matlab ODE solver until steady state (*t* = 10,000 hr). Starting from the steady state, we calculated equilibrium points by implementing a continuation method as described in Kuznetsov^41^. We calculated the eigenvalues to determine the stability of the system within the parameter space. For the A model (model equations are in the Supplementary Material), the initial conditions were *ϕ* = 0.13 and *μ* = 0.35, and the fixed parameters were *ε* = 0.1, *κ* = 5, *Γ*_1_′ = 1. For the R model, the initial conditions and parameter values are in Table 1, and we performed analysis with *Γ*_E_ on the interval [0, 0.007], and *Γ*_M_ on the interval [0, 0.5].

### Calculation of bifurcation points

We used Matlab and the MATCONT Matlab package^42^ to plot the bifurcation diagrams of the A and R models. The “continuation data” parameters we used were: maximum step-size of 1e-8 and the “correction data” parameter we used were: variable tolerance of 1e-8, function tolerance of 1e-8 and test tolerance of 1e-6.

### Parameter sensitivity analysis

The first order sensitivity


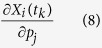


quantifies the effect of small perturbations in parameter values on the model variables. Equation (8) was calculated for each of the 33 model parameters (*j* = 1… 33, including *Γ*_M_, *Γ*_E_ and *S*), and for each of the 8 model variables (*i* = 1… 8). This involved numerically solving an augmented ODE system of dimension 272 (272 = 8 + 8*33), given by


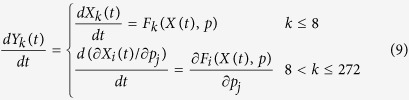


where *k* = 8 + (*j* − 1)*33 + *i* (*i* = 1…8; *j* = 1…33). In order to capture time-dependent parameter sensitivities, we integrated Eq. (9) over the time-interval *t* = 0 to *t* = 1,000 hr, at 1000 points (*k* = 1…1000). After solving the augmented ODE, we consolidated the time-sensitivities in a manner that takes into account possible noise in measurements^43^


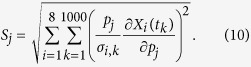


Here, *p*_j_ is the nominal parameter value, and *σ*_i,k_ = 1e-6 × *X*_i_(*t*_*k*_) + 1e-8. The relative and absolute tolerances we used in the ODE simulation were 1e-6 and 1e-8, respectively. The initial conditions used were in Table 1 for the model variables (*k* = 1… 8) and zero for all other variables (*k* > 8). The nominal parameter values are in Supplementary Table 4 and a Matlab code for parameter sensitivity analysis is provided in Supplementary File 1.

## Additional Information

**How to cite this article**: del Rosario, R. C. H. *et al.* MicroRNA inhibition fine-tunes and provides robustness to the restriction point switch of the cell cycle. *Sci. Rep.* **6**, 32823; doi: 10.1038/srep32823 (2016).

## Supplementary Material

Supplementary Information

Supplementary File 1

## Figures and Tables

**Figure 1:**
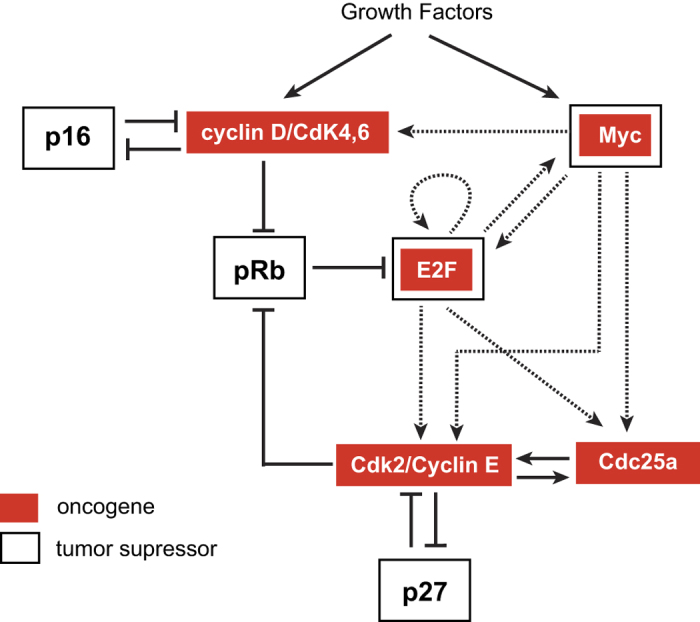
A cancer network commonly described as the Rb-E2F network. This is a network of oncogenes (red boxes) and tumor suppressor genes (white boxes) involved in the regulation of the Restriction Point^10^,^44^,^45^,^46^. The dashed arrow lines indicate transcriptional activation while solid hammer lines indicate inhibition. pRB, retinoblastoma protein; E2F, family of transcription factors consisting of E2F1, E2F2 and E2F3; p16, cyclin-dependent kinase inhibitor 2A; p27, cyclin-dependent kinase inhibitor 1B; cyclin D/Cdk4, 6: complex formed by cyclin D, Cdk4 and Cdk6; Cdk2/Cyclin E: complex formed by cyclin E and Cdk2; CdC25A, cell division cycle 25 homolog A.

**Table 1:** Parameter values and initial conditions of the variables of the R model.

*Parameter Values*			
*k*_*E*_ = 0.4 μM/hr	*k*_*CE*_ = 0.35 μM/hr	*d*_*μ*_ = 0.001/hr	*K*_*RP*_ = 0.01 μM
*k*_*M*_ = 1.0 μM/hr	*d*_*M*_ = 0.7/hr	*k*_*p1*_ = 18/hr	*K*_*Mμ*_ = 0.1 μM
*k*_*CD*_ = 0.03 μM/hr	*d*_*E*_ = 0.25/hr	*k*_*p2*_ = 18/hr	*K*_*Eμ*_ = 0.1 μM
*k*_*CDS*_ = 0.45 μM/hr	*d*_*CD*_ = 1.5/hr	*k*_*DP*_ = 3.6 μM/hr	*k*_*11a*_ = 0.05 μM/hr
*k*_*R*_ = 0.18 μM/hr	*d*_*CE*_ = 1.5/hr	*K*_*M*_ = 0.15 μM	*k*_*11b*_ = 0.05 μM/hr
*k*_*RE*_ = 180 μM/hr	*d*_*R*_ = 0.06/hr	*K*_*E*_ = 0.15 μM	*k*_*11c*_ = 0.0 μM/hr
*k*_*b*_ = 0.003 μM/hr	*d*_*RP*_ = 0.06/hr	*K*_*CD*_ = 0.92 μM	
*K*_*S*_ = 0.5 μM/hr	*d*_*RE*_ = 0.03/hr	*K*_*CE*_ = 0.92 μM	
***Initial Conditions***			
*M* = 0 μM	*CD* = 0 μM	*R* = 0 μM	*RE* = 0.55 μM
*E* = 0 μM	*CE* = 0 μM	*RP* = 0 μM	*μ* = 0 μM
