# Supplementary material for: MicroRNA inhibition fine-tunes and provides robustness to the restriction point switch of the cell cycle
Source: Sci Rep. 2016 Sep 9;6:32823. doi: 10.1038/srep32823 (PMC5017317; doi:10.1038/srep32823)
Supplement: Supplementary Information [file srep32823-s1.pdf]

MicroRNA inhibition fine-tunes and provides robustness to the restriction point switch of the cell cycle

Ricardo C.H. del Rosario[1,*], Joseph Ray Clarence G. Damasco[2], Baltazar D. Aguda[3,*]

1. Stanley Center for Psychiatric Research, Broad Institute of MIT and Harvard, Cambridge MA 02142, USA
2. Institute of Mathematics, University of the Philippines, Diliman, Quezon City 1101 Philippines
3. Philippine Genome Center, University of the Philippines, Diliman, Quezon City 1101 Philippines

*Correspondence and requests for material should be sent to: R.C.H.d.R (email: rcdelros@broadinstitute.org) or B.D.A (email: bdaguda@up.edu.ph).

SUPPLEMENTARY MATERIAL

Noise Amplification

We provide the elements of the matrices that are involved in the linear system that arises from the steady-state normalized FDT[39]

$$M\eta + \eta M^T + D = 0.$$

The components of the matrices η, M and D are given by

$$\eta_{ij} = \frac{\sigma_{ij}}{X_i X_j}, M_{ij} = \frac{X_j}{X_i} A_{ij}, D_{ij} = \frac{B_{ij}}{X_i X_j}, i, j = 0,\ldots,8.$$

The elements A_{ij} come from the *elasticities*[31, 39, 40],

$$A_{ij} = -\frac{F_i}{X_j} \frac{\partial \ln\left(F_i\left(X_0, X_1,\ldots,X_8\right)\right)}{\partial \ln\left(X_j\right)}, i = 1,\ldots,8, j = 0,\ldots,8$$

$$A_{0j} = \begin{cases} 1/\tau_0 & j = 0 \\ 0 & j \neq 0. \end{cases}$$

B is the diffusion matrix[31, 39, 40] with elements

$$B_{ij} = \sum_k v_{jk} v_{ik} R_k, i = 1,\ldots,8, j = 0,\ldots,8,$$

where the reaction k occurs with rate R_k and produces v_{ik} molecules of species i. Thus,

$$B = \begin{bmatrix} 2/(\tau_0 X_0) & 0 & 0 & 0 & 0 & 0 & 0 & 0 & 0 \\ 0 & b_{11} & 0 & 0 & 0 & 0 & 0 & 0 & 0 \\ 0 & 0 & b_{22} & 0 & 0 & 0 & b_{27} & 0 & 0 \\ 0 & 0 & 0 & b_{33} & 0 & 0 & 0 & 0 & 0 \\ 0 & 0 & 0 & 0 & b_{44} & 0 & 0 & 0 & 0 \\ 0 & 0 & 0 & 0 & 0 & b_{55} & b_{56} & b_{57} & 0 \\ 0 & 0 & 0 & 0 & 0 & b_{65} & b_{66} & b_{67} & 0 \\ 0 & 0 & b_{72} & 0 & 0 & b_{75} & b_{76} & b_{77} & 0 \\ 0 & 0 & 0 & 0 & 0 & 0 & 0 & 0 & b_{88} \end{bmatrix}$$

where

$$b_{11} = v_1 + d_M X_1 \qquad b_{65} = b_{56}$$
$$b_{22} = v_3 + v_7 + v_8 + d_E X_2 \qquad b_{66} = v_7 + v_9 + v_{10} + d_{RP} X_6$$
$$b_{27} = v_7 + v_8 \qquad b_{67} = v_7$$
$$b_{33} = v_2 + v_5 + d_{CD} X_3 \qquad b_{72} = b_{27}$$
$$b_{44} = v_4 + d_{CD} X_4 \qquad b_{75} = b_{57}$$
$$b_{55} = v_6 + v_{10} + v_8 + v_9 + d_R X_5 \qquad b_{76} = b_{67}$$
$$b_{56} = v_9 + v_{10} \qquad b_{77} = v_8 + v_7 + d_{RE} X_7$$
$$b_{57} = v_8 \qquad b_{88} = v_{11} + d_\mu X_8.$$

The parameter τ_0 is the average lifetime of X_0. We used the value 0.1 in our calculations, but this value did not affect the qualitative trends we showed in Fig. 5 and Supplementary Figs. 2-6.

The model of Aguda *et al.* (2008)

The two-dimensional A model represents a simplified microRNA regulatory network involving miR-17-92 and the transcription factors c-Myc and E2F. The dimensionless kinetic equations are given below, where ϕ is the dimensionless concentration of the protein p, and μ is the dimensionless concentration of the miR cluster m

$$\varepsilon \frac{d\phi}{dt} = \alpha' + \left(\frac{\kappa \phi^2}{\Gamma_1' + \phi^2 + \Gamma_2' \mu} \right) - \phi$$

$$\frac{d\mu}{dt} = 1 + \phi - \mu,$$

with non-dimensionalized parameters

$$\phi = \left(\frac{k_2}{\beta} \right) p, \quad \mu = \left(\frac{\gamma}{\beta} \right) m, \quad \tau = \gamma t$$

$$\varepsilon = \frac{\gamma}{\delta}, \quad \alpha' = \left(\frac{k_2}{\delta \beta} \right) \alpha, \quad \kappa = \frac{k_1 k_2}{\delta \beta}.$$

$$\Gamma_1' = \left(\frac{k_2^2}{\beta^2} \right) \Gamma_1 \quad \Gamma_2' = \left(\frac{k_2^2}{\beta \gamma} \right) \Gamma_2.$$

Supplementary Table 1. Number of cells in the on and off states in Figure 4. MiRNA strengths: 1 ($\Gamma_E = \Gamma_M$=0; no miR); 2 (Γ_E=6.3e-4, Γ_M=0.0404); 3 (Γ_E=6.3e-4, Γ_M =0.141); 4 (Γ_E=6.3e-4, Γ_M=1.8).

	No. Cells with E2F < 0.01				No. of Cells withE2F > 1.0			
**miRNA strength**	**1**	**2**	**3**	**4**	**1**	**2**	**3**	**4**
No pulse, S=0.8	4360	4526	4755	9188	3144	2720	1982	4
With pulse, S=0.8	254	275	296	7120	5705	5009	3759	12
No pulse, S=1.0	1232	1303	1413	4741	5356	4844	3839	101
With pulse, S=1.0	43	45	50	2719	6163	5608	4476	107

Supplementary Table 2. Summary of stochastic simulation results. The descriptive statistics of the E2F values from 10,000 simulations are given for each of the plots in Fig. 4.

Pulse	Final S	Γ_E	Γ_M	Average E2F	25th Percentile E2F	Median E2F	75th Percentile E2F
No	0.8	0	0	5.90e-01	1.82e-03	6.14e-01	1.10e+00
No	0.8	6.3e-4	0.0404	5.46e-01	1.73e-03	5.11e-01	1.04e+00
No	0.8	6.3e-4	0.141	4.69e-01	1.60e-03	9.99e-02	9.12e-01
No	0.8	6.3e-4	1.8	1.41e-02	9.00e-04	1.29e-03	3.00e-03
Yes	0.8	0	0	9.65e-01	7.48e-01	1.03e+00	1.28e+00
Yes	0.8	6.3e-4	0.0404	9.18e-01	7.01e-01	9.86e-01	1.23e+00
Yes	0.8	6.3e-4	0.141	8.33e-01	6.12e-01	8.95e-01	1.14e+00
Yes	0.8	6.3e-4	1.8	1.46e-01	2.40e-03	1.18e-02	2.03e-01
No	1.0	0	0	1.06e+00	8.29e-01	1.06e+00	1.30e+00
No	1.0	6.3e-4	0.0404	1.00e+00	7.77e-01	1.00e+00	1.24e+00
No	1.0	6.3e-4	0.141	8.95e-01	6.72e-01	8.97e-01	1.12e+00
No	1.0	6.3e-4	1.8	4.30e-02	2.41e-03	4.78e-03	1.20e-02
Yes	1.0	0	0	1.11e+00	8.83e-01	1.09e+00	1.33e+00
Yes	1.0	6.3e-4	0.0404	1.06e+00	8.41e-01	1.05e+00	1.27e+00
Yes	1.0	6.3e-4	0.141	9.75e-01	7.55e-01	9.60e-01	1.18e+00
Yes	1.0	6.3e-4	1.8	2.00e-01	8.91e-03	4.03e-02	3.52e-01

Supplementary Table 3. Range of parameter values used in noise susceptibility and noise amplification analysis. These are the range of parameter values used to plot Fig. 5 and Supplementary Figs. 2-6. The values of the other parameters not indicated below are found in Table 1.

	S	Γ_E	Γ_M	k_E
Fig. 5	[1.47e-1, 2.47]	[0, 7.2e-3]	[0, 5e-2]	0.4
Supp. Fig. 2	[1.47e-1, 4.21]	[0, 3e-3]	[0, 0.8]	0.4
Supp. Fig. 3	[1.80e-1, 6.25e-1]	[0, 2e-3]	0	[0.25, 0.35]
Supp. Fig. 4	[2.28e-1, 1.28]	0	[0, 9e-2]	[0.2, 0.3]
Supp. Fig. 5	[2.28e-1, 2.21]	0	[0, 0.4]	[0.25, 0.3]
Supp. Fig. 6	[2.28e-1, 6.25e-1]	[0, 2e-3]	0	[0.25, 0.3]

Supplementary Table 4. Parameters used in Parameter Sensitivity Analysis. Combinations of these parameters yield 3,168 parameter sets used to plot Supplementary Fig. 7. All other parameter values are given in Table 1.

**Parameter**	**Values**
S	0.8, 1.0, 2.0, 3.0, 4.0, 5.0
Γ_E	0, 6.36e-4, 7.5e-4, 1.5e-3, 2.05e-3, 3e-3, 4.88e-3, 6.93e-3
Γ_M	0, 1.25e-6, 2.5e-2, 3.75e-2, 4.04e-2, 1.41e-1, 0.2, 0.4, 0.6, 0.8, 1.8
k_E	0.125, 0.263, 0.274, 0.287, 0.3, 0.35

Supplementary Figures

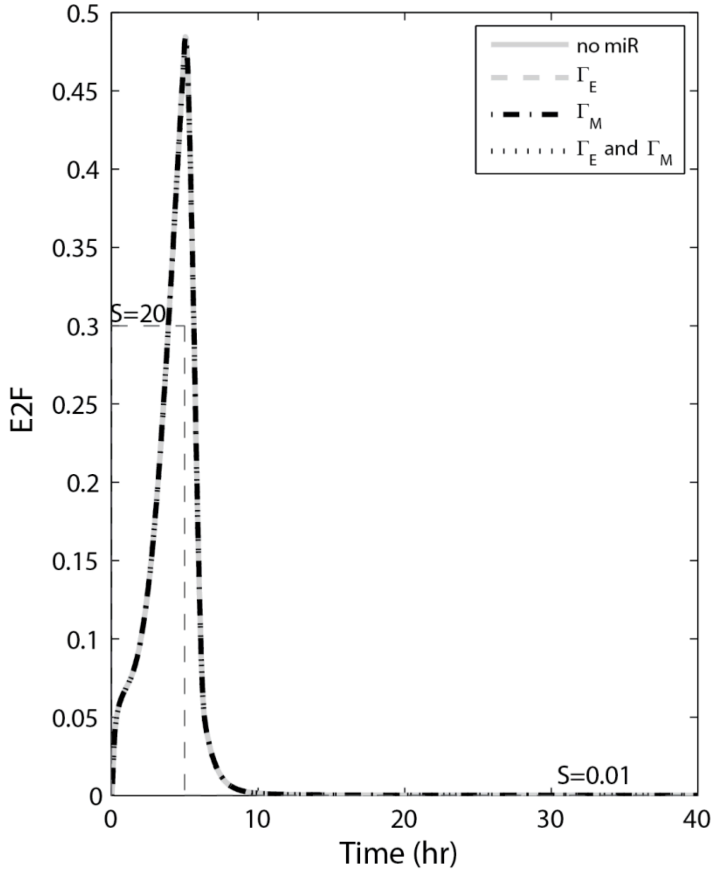

Supplementary Figure 1. The R-model is excitable. A serum pulse (*S*=20 for 5 hours) causes the system to initially proceed to the on state (the on state is E2F>0.01, see Fig. 3b), but rapidly goes to the off state after the pulse was removed (final *S*=0.01). Grey solid lines (no miR): Γ_M=0, Γ_E=0; Dashed grey lines (Γ_E): Γ_M=0, Γ_E=6.3e-4; Black dash-dot lines (Γ_M): Γ_M=0.141, Γ_E=0; Black dotted lines (Γ_E and Γ_M): Γ_M=0.141, Γ_E=6.3e-4.

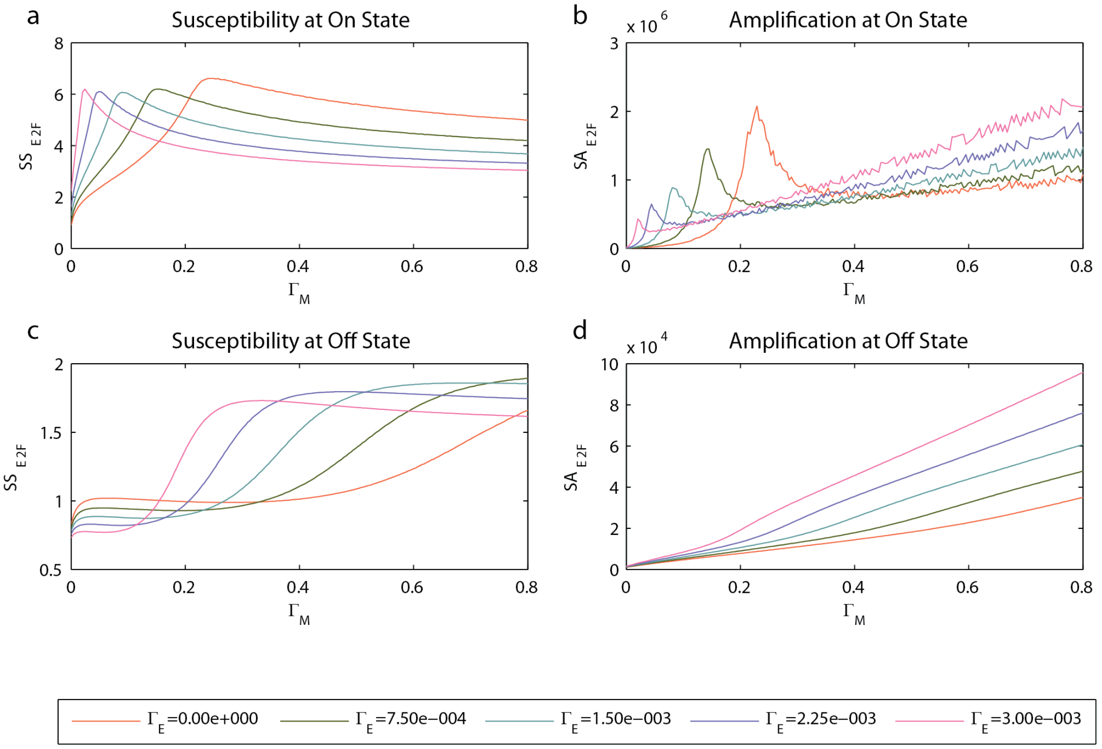

Supplementary Figure 2. Noise amplification (*SA*) and noise susceptibility (*SS*) curves for E2F as parameters Γ_M and Γ_E vary. **(a)** Noise susceptibility at the on state. **(b)** Noise amplification at the on state. **(c)** Noise susceptibility at the off state. **(d)** Noise amplification at the off state. 5 different values of parameter Γ_E were used (legend bar) and the values of the other parameters are given in Table 1. Initial conditions that lead to the on and off states were chosen, and the model values at the steady states were used in calculating noise amplification and susceptibility (Methods).

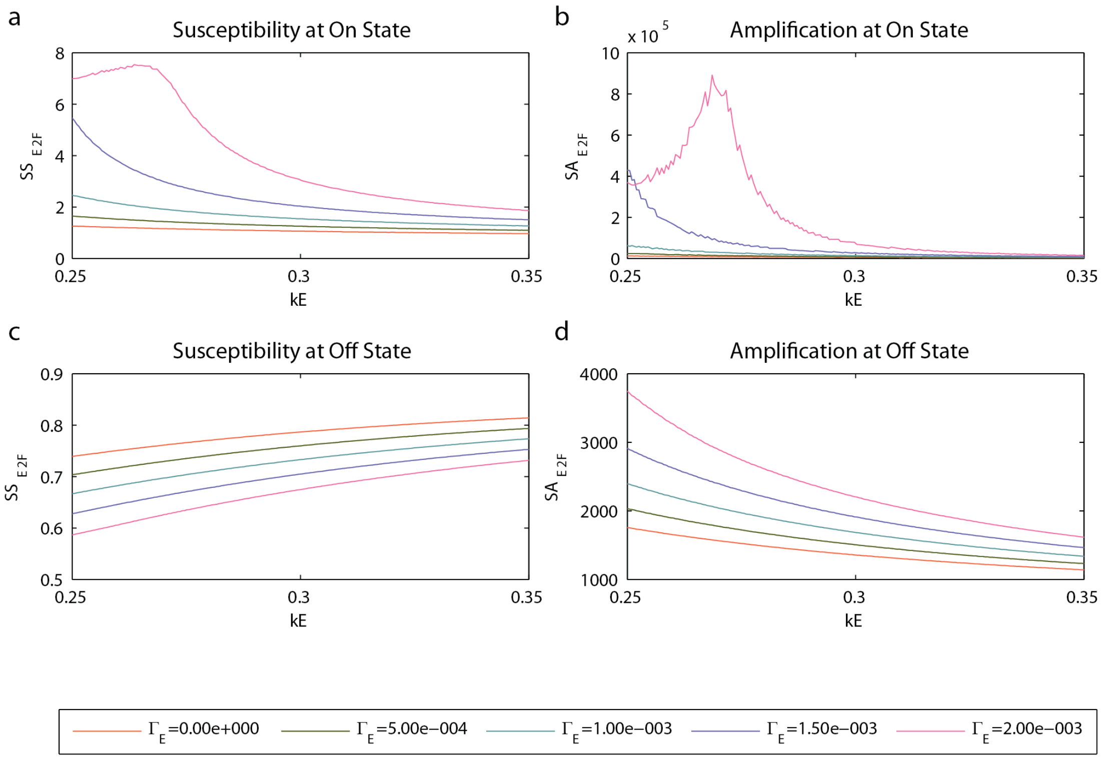

Supplementary Figure 3. Noise amplification (*SA*) and noise susceptibility (*SS*) curves for E2F as parameters Γ_E and k_E vary. **(a)** Noise susceptibility at the on state. **(b)** Noise amplification at the on state. **(c)** Noise susceptibility at the off state. **(d)** Noise amplification at the off state. 5 different values of parameter Γ_E were used (legend bar) and the values of the other parameters are given in Table 1. Initial conditions that lead to the on and off states were chosen, and the model values at the steady states were used in calculating noise amplification and susceptibility (Methods).

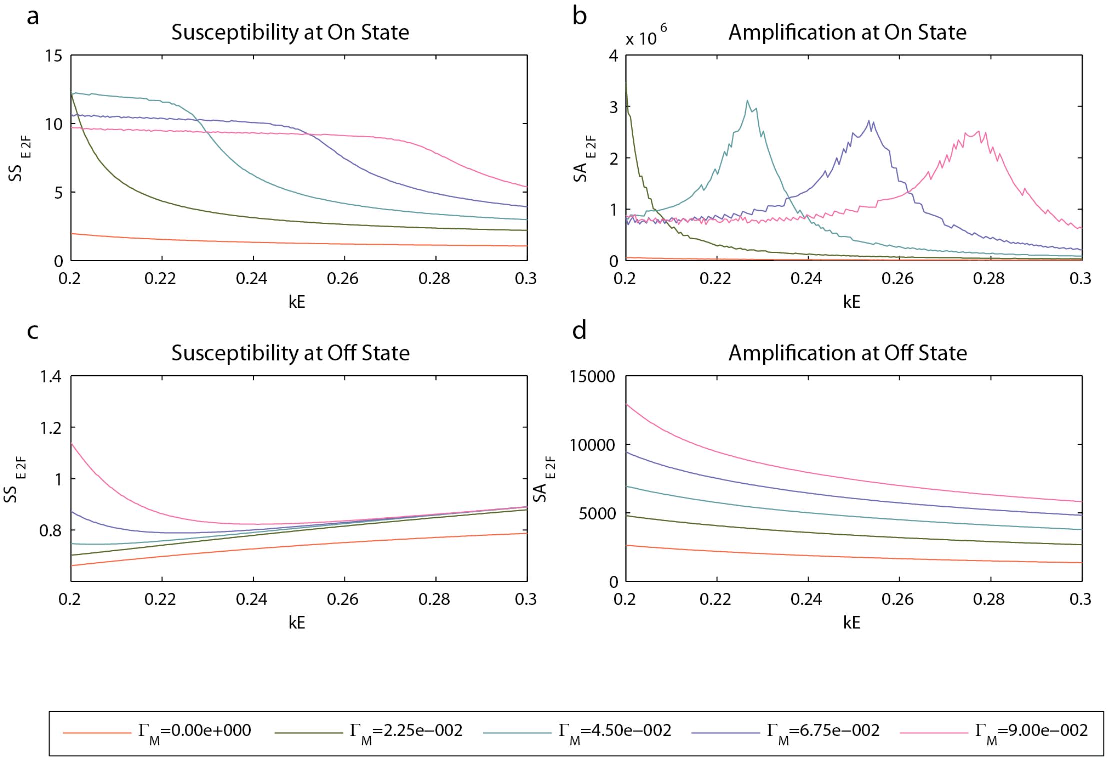

Supplementary Figure 4. Noise amplification (*SA*) and noise susceptibility (*SS*) curves for E2F as parameters Γ_{M} and k_{E} vary. **(a)** Noise susceptibility at the on state. **(b)** Noise amplification at the on state. **(c)** Noise susceptibility at the off state. **(d)** Noise amplification at the off state. 5 different values of parameter Γ_{M} were used (legend bar) and the values of the other parameters are given in Table 1. Initial conditions that lead to the on and off states were chosen, and the model values at the steady states were used in calculating noise amplification and susceptibility (Methods).

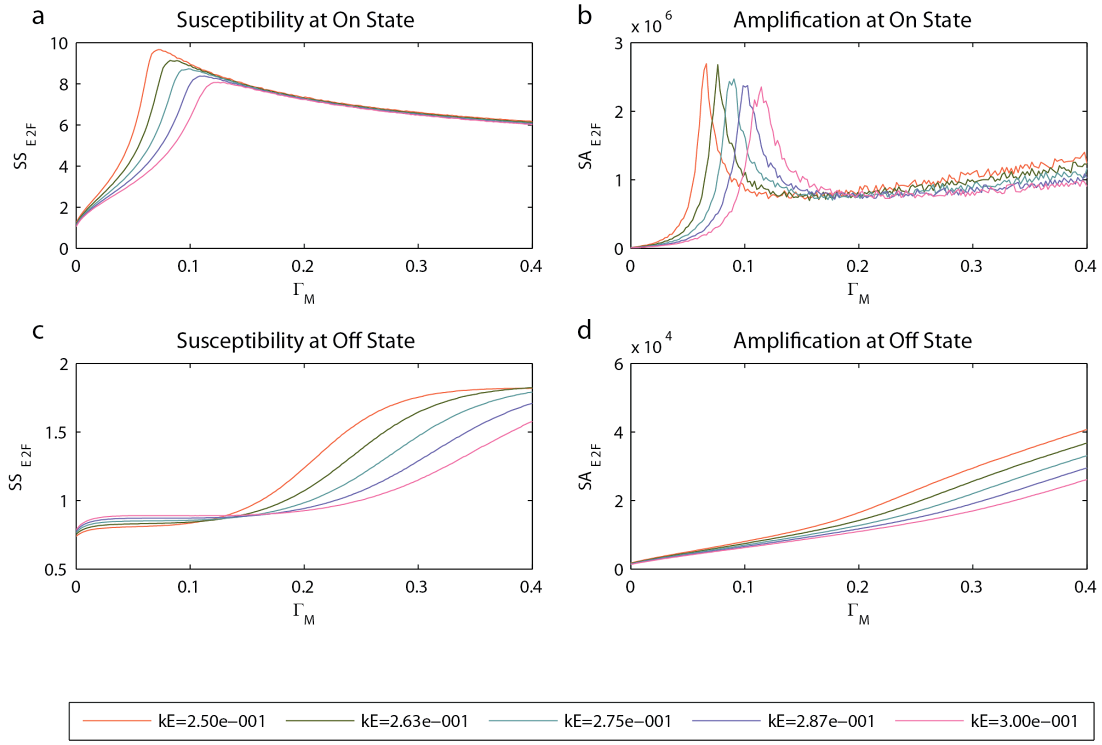

Supplementary Figure 5. Noise amplification (*SA*) and noise susceptibility (*SS*) curves for E2F as parameters k_E and Γ_M vary. **(a)** Noise susceptibility at the on state. **(b)** Noise amplification at the on state. **(c)** Noise susceptibility at the off state. **(d)** Noise amplification at the off state. 5 different values of parameter k_E (legend bar) were used and the values of the other parameters are given in Table 1. Initial conditions that lead to the on and off states were chosen, and the model values at the steady states were used in calculating noise amplification and susceptibility (Methods).

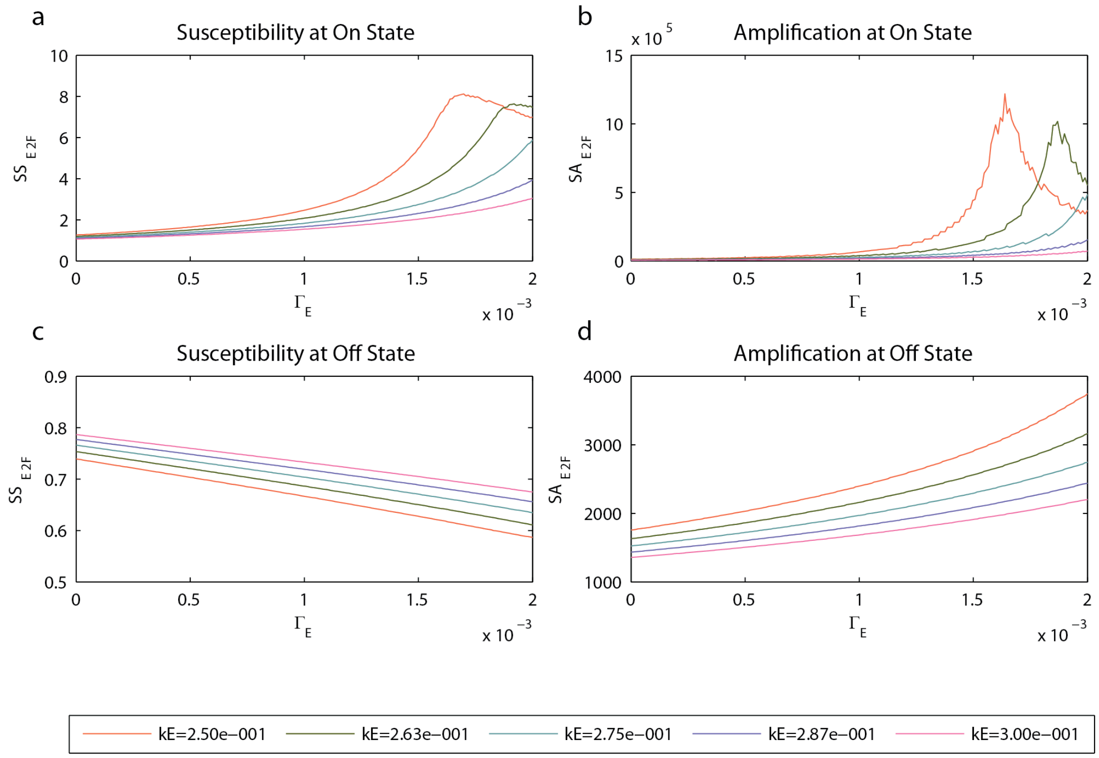

Supplementary Figure 6. Noise amplification (*SA*) and noise susceptibility (*SS*) curves for E2F as parameters k_E and Γ_E vary. **(a)** Noise susceptibility at the on state. **(b)** Noise amplification at the on state. **(c)** Noise susceptibility at the off state. **(d)** Noise amplification at the off state. 5 different values of parameter k_E (legend bar) were used and the values of the other parameters are given in Table 1. Initial conditions that lead to the on and off states were chosen, and the model values at the steady states were used in calculating noise amplification and susceptibility (Methods).

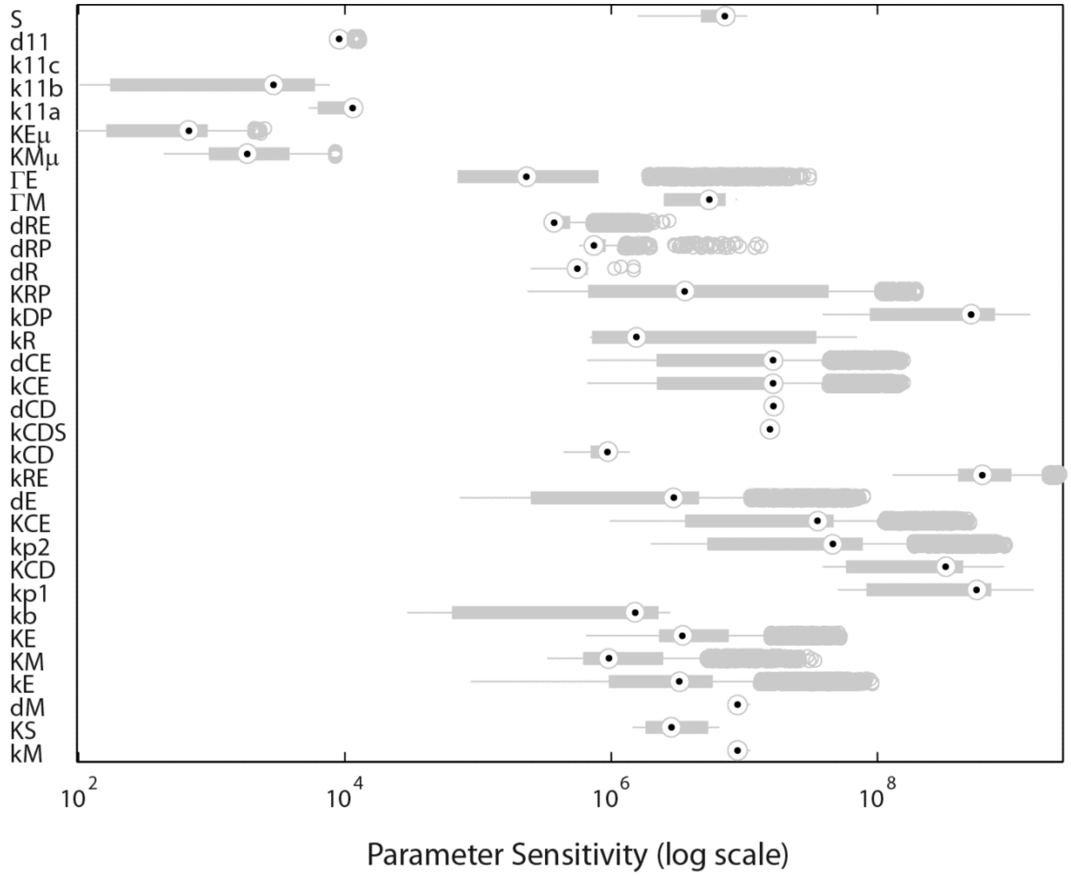

Supplementary Figure 7. Box plots of time-dependent parameter sensitivities for each of the 33 model parameters. 3,168 parameter sets were used and the parameter sensitivity (Eq. (11) in main text) was calculated for each. The dark circles indicate the median. Nominal parameter values are in Supplementary Table 4.
